# Digital gene expression analysis of two life cycle stages of the human-infective parasite, *Trypanosoma brucei gambiense *reveals differentially expressed clusters of co-regulated genes

**DOI:** 10.1186/1471-2164-11-124

**Published:** 2010-02-22

**Authors:** Nicola J Veitch, Paul CD Johnson, Urmi Trivedi, Sandra Terry, David Wildridge, Annette MacLeod

**Affiliations:** 1 Wellcome Centre for Molecular Parasitology, Glasgow Biomedical Research Centre, University of Glasgow, Glasgow, G12 8TA, UK; 2 Robertson Centre for Biostatistics, Boyd Orr Building, University of Glasgow, Glasgow, G12 8QQ, UK; 3 The Gene Pool, Ashworth Laboratories, The King's Buildings, The University of Edinburgh, Edinburgh, EH9 3JT, UK

## Abstract

**Background:**

The evolutionarily ancient parasite, *Trypanosoma brucei*, is unusual in that the majority of its genes are regulated post-transcriptionally, leading to the suggestion that transcript abundance of most genes does not vary significantly between different life cycle stages despite the fact that the parasite undergoes substantial cellular remodelling and metabolic changes throughout its complex life cycle. To investigate this in the clinically relevant sub-species, *Trypanosoma brucei gambiense*, which is the causative agent of the fatal human disease African sleeping sickness, we have compared the transcriptome of two different life cycle stages, the potentially human-infective bloodstream forms with the non-human-infective procyclic stage using digital gene expression (DGE) analysis.

**Results:**

Over eleven million unique tags were generated, producing expression data for 7360 genes, covering 81% of the genes in the genome. Compared to microarray analysis of the related *T. b. brucei *parasite, approximately 10 times more genes with a 2.5-fold change in expression levels were detected. The transcriptome analysis revealed the existence of several differentially expressed gene clusters within the genome, indicating that contiguous genes, presumably from the same polycistronic unit, are co-regulated either at the level of transcription or transcript stability.

**Conclusions:**

DGE analysis is extremely sensitive for detecting gene expression differences, revealing firstly that a far greater number of genes are stage-regulated than had previously been identified and secondly and more importantly, this analysis has revealed the existence of several differentially expressed clusters of genes present on what appears to be the same polycistronic units, a phenomenon which had not previously been observed in microarray studies. These differentially regulated clusters of genes are in addition to the previously identified RNA polymerase I polycistronic units of variant surface glycoproteins and procyclin expression sites, which encode the major surface proteins of the parasite. This raises a number of questions regarding the function and regulation of the gene clusters that clearly warrant further study.

## Background

All organisms are capable of adapting to their environment, which is usually achieved by adjusting gene expression levels, often at transcription initiation. It has been the goal of many studies to determine the changes in transcription in response to varying environmental conditions, such as when the cells are under stress, drug pressure, in different environments or subject to immune responses. Traditional genome-wide analysis of gene expression of cells under different conditions or, in the case of parasites, at different life cycle stages, has mainly been carried out by hybridization-based methods such as microarrays [1-5]. Such hybridization-based approaches are subject to non-specific hybridization, cross hybridization and nonlinear and saturable hybridization kinetics, providing relative rather than direct quantitative expression levels that are not likely to be comparable between experiments[6].

Two alternative approaches to gene expression analysis are sequence based and have become increasingly popular due to recent developments in sequencing technologies[7]. The first is the direct sequencing of cDNA, termed RNAseq [8-11]. The second approach is based on sequencing serial analysis of gene expression (SAGE) libraries (termed digital gene expression (DGE)), a method that generates a digital output proportional to the number of transcripts per mRNA[12,13]. These methods are limited only by the depth of coverage of the sequencing undertaken and both approaches have the benefit of not requiring presynthesised oligonucleotide probes (as in microarrays), allowing the direct enumeration of transcript molecules, i.e. digital quantification, which is directly comparable across different experiments.

The SAGE/DGE approach (outlined in additional file 1) is based on the addition of specific adapters to poly(A) cDNA, which has been digested with a restriction enzyme, typically *Nla*III. Further digestion of the cDNA with an enzyme that recognises within the adapter but cleaves 21 bp downstream generates a 21 base tag from that transcript and unlike traditional SAGE, where the tags are ligated together and then sequenced (hence the term 'serial'), the tags are directly sequenced using next generation sequencing technology, allowing direct quantification of the number of transcripts in a sample. This process will generate a sequence tag from transcripts that contain *Nla*III sites, preferentially from the 3' end of the transcript. This is typically either in the 3' untranslated regions (UTRs) or the 3' end of the open reading frame (ORF). Transcription profiling using the SAGE and more recently the DGE method has been used extensively to compare expression profiles in a range of tissues [14-24]. The advantage of DGE is the larger dynamic range obtained per experiment. However, there are a number of disadvantages with the DGE approach. It does not generate the added value of determining 5' and 3' UTRs or alternative splice variants, which is an important consideration for the analysis of most organisms, (less so for trypanosomes, which have very few cis-spliced genes[25]). DGE also requires a reference transcriptome for comparison and is confined to the analysis of genes containing the four-base restriction site (typically *Nla*III) used in the library construction. If the 5' and 3' UTRs of genes are not included in the reference transcriptome, transcripts with *Nla*III sites in the 3'UTRs or within 21 bp of the stop codon will not be represented. Despite only obtaining a partial transcriptome of the organism under investigation, DGE is a useful tool in the analysis of gene expression at an unprecedented level of sensitivity, particularly when comparing two very similar samples. This sensitive and cost-effective approach can be applied to the study of gene expression in cells under a spectrum of experimental or natural conditions. One group of organisms that is subjected to dramatic environmental changes throughout their life cycle, including larges changes in temperature, nutrients and host immune defences is the parasitic protozoa.

The evolutionarily ancient single-celled parasite *Trypanosoma brucei gambiense *has a particularly complicated life cycle, passing between the tsetse vector and different mammalian hosts species including humans, where it causes a fatal disease in humans, African sleeping sickness[26,27]. Throughout its life cycle, the parasite has to adapt to extreme changes in its environment, in response to which it differentiates into several morphologically distinct forms, involving organelle repositioning and changes to its metabolism[27]. To what extent are these gross morphological and metabolic changes controlled by alterations in the transcriptome of the parasites between different life cycle stages?

Studies to date investigating the transcriptome of African trypanosomes have focused mainly on the non-human infective sub-species, *T. b. brucei*, revealing that transcription in trypanosomes is unusual. The majority of trypanosome genes are transcribed by RNA polymerase II in long polycistronic units, which are then processed by trans-splicing that adds a 39-nucleotide spliced leader to every mRNA to produce monocistronic mature mRNAs [28-30]. However evidence to date suggests that, unlike bacterial operons, most genes in polycistronic units are not functionally related and are not co-regulated. The steady state levels of mRNA appear to be determined predominantly post-transcriptionally by mRNA degradation controlled by the 3'UTR of transcripts[31]. There are notable exceptions to this pattern of gene regulation in that some gene arrays that encode the parasite's variant surface glycoproteins (VSGs) and procyclin expressions sites in bloodstream forms and procyclic forms, respectively, are co-regulated and co-transcribed by RNA polymerase I[32]. This raises the question, are there other co-regulated polycistronic units within the genome? If so, when are they expressed and what do they encode? Are co-regulated polycistronic units transcribed by RNA polymerase I alone? While many genes in the genome appear to be arranged in the same orientation and are presumably transcribed as polycistronic units, several small open reading frames (ORFs), typically between 150-450 bp in size, with the opposite orientation have been annotated in the genome as being 'unlikely'[33]. Do these small ORFs represent a novel class of trypanosome genes that are transcribed in the opposite orientation from other genes in the polycistron or are they an artefact of gene prediction software? In order to address these questions an analysis of the transcript abundance of these ORFs is required.

Microarray analysis has been carried out to examine changes in transcript abundance between the mammalian bloodstream forms and the insect procyclic forms of the related parasite *T. b. brucei *and the results suggest that relatively few parasite genes are differentially regulated between the two life cycle stages[2,5,34]. In this study we add to the transcriptome analysis of trypanosomes, demonstrating the use of DGE to examine the transcriptome of two life cycle stages of the clinically relevant sub-species, *T. b. gambiense*.

## Results and Discussion

### Library production and DGE tag annotation

In this study we determined and compared the partial transcriptome of two different life cycle stages of the pleomorphic *T. b. gambiense *strain, STIB 386; the procyclic (insect) stage, which is not human-infective, and the potentially human-infective bloodstream stage. RNA from parasites of both life cycle stages was prepared from exponentially growing cells. Three DGE libraries were made from replicate cultures for each life cycle stage and sequenced using Solexa (Illumina) technology. The three procyclic form libraries (termed procyclic A-C) and three bloodstream form libraries (termed bloodstream A, C and D) generated 5.97 × 10^6 ^and 5.54 × 10^6 ^tags, respectively, for the two classes of samples, after filtering for poor quality sequencing scores (Table 1). The normalized tag counts are presented as supplementary data (Additional file 2) and all raw data is available from gene expression omnibus (GEO) accession number GSE18065 and will be made available on TritrypDB[35]. The number of tags generated using this methodology is at least 300-fold greater than the number of tags obtained for a *T. b. brucei *strain, using SAGE with traditional sequencing methods[36].

**Table 1:** A comparison of the number of digital tags generated from the *T. b. gambiense *procyclic and bloodstream libraries.

Libraries	Total tags	Aligned tags	% aligned tags	No. unique genes hit
BSFA	2,466,628	883,737	36	7,171
BSFC	1,218,797	279,936	23	7,062
BSFD	1,860,336	612,444	33	6,020
PCFA	2,831,525	699,474	27	7,237
PCFB	2,180,993	592,429	27	7,139
PCFC	966,319	110,500	11	6,782

The tags were aligned to the fully annotated reference transcriptome of *T. b. brucei*, TREU 927[35] allowing for a two base pair mismatch for any polymorphisms between the reference genome and the genome of STIB 386. As the 5' and 3' UTR of most *T. b. brucei *genes have not be defined, the annotated transcriptome contained only ORFs, limiting this analysis to tags that align uniquely to the ORF of genes. The reference transcriptome represented the chromosome internal genes and did not extend past the sub-telomeric regions of the chromosomes or include the minichromosomes. The number of tags that were of good quality and aligned uniquely to the reference genome for each library is presented in Table 1, providing expression data for 7,360 genes. However there were a large number of tags that either aligned to multiple sites or no sites within the annotated reference transcriptome, in part reflecting the incomplete nature of the *in silico *transcriptome and the distribution of *Nla*III sites in the genome. It is likely that many of the tags that did not align to the transcriptome actually aligned to the 3'UTR of transcripts as genes in the *T. brucei *genome have relatively long 3'UTRs with a median size of 348 nt[37].

The dynamic range of DGE spanned four orders of magnitude. The most abundant transcript in all procyclic form libraries was that for the heat shock gene, HSP83 (Tb10.26.1080), with a maximum tag count in procyclic form library A of 22,977 tags accounting for 3.3% of uniquely aligned tags for that sample. However, the tag counts for the majority of genes were low in both the bloodstream and procyclic forms.

### Reproducibility

**Figure 1:**
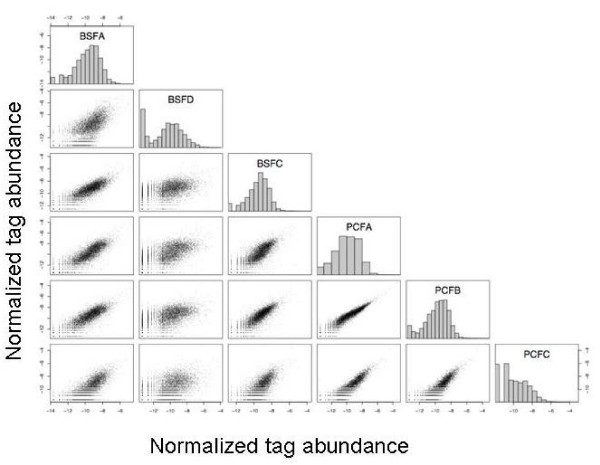
**Scatterplot showing the correlation between the expression levels for each gene for each library**. Scatterplots of the normalised tag abundance (transformed using log_e_(tag abundance + 1)) for each library in all pairwise combinations. Histograms show the distributions of the transformed normalised abundances.

Digital gene expression has been shown to have very low technical variability[38]. In order to examine the ability of DGE to detect subtle differences between biological replicates, DGE libraries generated from RNA extracted from three identical cultures grown under the same conditions for each life cycle stage were compared. The number of tags generated for each library was similar (Table 1). Figure 1 shows a series of scatter plots comparing the normalised tag counts (log transformed) for the replicate libraries in each pairwise combination. A high correlation was observed between replicates, indicating a high degree of reproducibility of technical and biological replicates, with an average Pearson's correlation coefficient of r = 0.898 for the procyclic form replicates and r = 0.843 for the bloodstream form replicates following log transformation (Figure 1). Bloodstream form parasites when grown at high density are capable of differentiating into the next life cycle stage, the short stumpy form, which is cell cycle arrested and pre-adapted to life in the tsetse fly[27]. The inability to synchronise pleomorphic cells could result in the presence in some cultures of short stumpy differentiated forms, resulting in reduced reproducibility in bloodstream form cultures compared to procyclic forms. Although no short stumpy forms were observed in any of the bloodstream form cultures, and the overall correlation coefficient for bloodstream form replicates was reasonably high, similar to that for procyclic forms, a small proportion of trypanosomes could have begun to differentiate into stumpy forms. Indeed, PAD1, a gene previously found to be enriched in stumpy forms over slender bloodstream forms and procyclics, was up-regulated 5-fold in the bloodstream forms in this analysis, although not statistically significant at the 0.2 false discovery rate (FDR) (see following section). This indicated that some of the bloodstream form cells used in this analysis may have begun the differentiation process to stumpy forms. The variation between replicate cultures, although small, clearly demonstrated the value of performing biological replicates.

### Differentially expressed genes

The expressed tags that uniquely aligned to the reference transcriptome generated expression data for 7,360 genes, approximately 81% of the total number of genes (n = 9,068) in the annotated genome[33] (Additional file 2). Using a threshold of 2.5 average fold change in expression for comparison with previous studies using microarrays, 1,933 genes were up-regulated in bloodstream forms and only 153 genes up-regulated in procyclic forms, a total of 28% differentially expressed genes (Additional file 3), compared to 2% using microarray analysis [2,5,34]. However, this difference was less an indication of biological significance than a product of the arbitrary nature of using a fold-change cut-off, which was insensitive to differences in factors affecting statistical power such as technical sensitivity, sample size and variability among biological replicates. In this analysis we have employed a statistically valid method of identifying differentially expressed genes taking into account biological replicates, termed Rank Products[39], which ranked genes by degree of differential expression and estimated both gene-specific *P*-values and a FDR that allowed many hypotheses to be tested simultaneously. Intuitively, setting a low, stringent FDR would define a smaller but better supported set of genes. Using a highly stringent FDR of 0.1, where 10% of identified genes would be expected to be false positives, a total of 73 genes were up-regulated in bloodstream forms compared to procyclics and 25 genes were up-regulated in procyclic forms compared to bloodstream forms. At a slightly less stringent FDR of 0.2, 126 genes were up-regulated in bloodstream forms and 63 genes were up-regulated in procyclic forms (Additional file 4). The overall difference in regulation is illustrated by a quantile-quantile (QQ) plot (Additional file 5) where the observed -log_10_(*P*-value) for each gene was plotted against the expected value under the null hypothesis of no difference in gene expression between life cycle stages. The observed values for both bloodstream and procyclic stages rose above the y = x line at low *P-*values (approx. P < 0.1, or -log10(P) > 1) indicating that there were many more low *P*-values than expected by chance, i.e. many genes were strongly differentially expressed, with more genes differentially expressed in bloodstream stages than in procyclic stages. Throughout this analysis all genes with differential gene expression are referred to as being up-regulated either in bloodstream or procyclics. However this does not imply a regulatory mechanism merely a comparative description that could have been generated by a down-regulation in one of the life-cycle stages.

**Figure 2:**
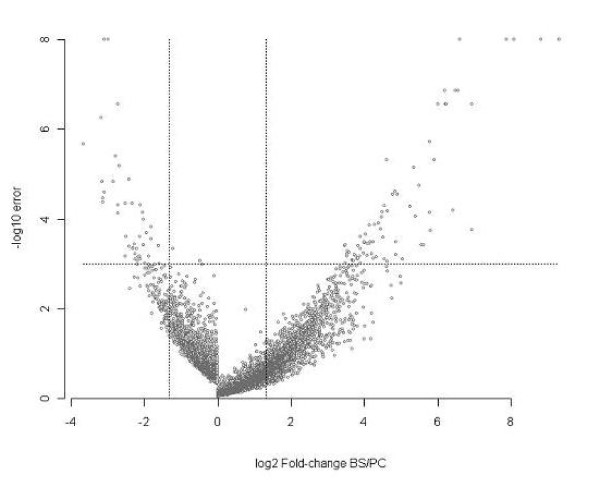
**Volcano plot of aligned tags**. For every tag the ratio in expression levels in the bloodstream form libraries over that in the procyclic libraries is plotted against the -log error rate. The horizontal line indicates the significance threshold applied (0.1 FDR) and the vertical lines indicate the 2.5-fold change threshold.

Remarkably the fold change of many of these differentially expressed genes was extremely high (Additional file 3), ranging from 11 to 634-fold up-regulated in bloodstream forms and 3.5 to 12.6 up-regulated in procyclic forms. The distribution of the detected fold change is illustrated in the Volcano plot (Figure 2), where the statistical significance of each gene was plotted against fold change. The plot shows that the tags with the highest average differences between life cycle stages (far right and left of the plot) also showed the greatest degree of significance. Another feature of the plot was the tendency of procyclics to show more significant differences than bloodstream forms at fold changes close to zero, reflecting the greater reproducibility within procyclics leading to higher statistical power relative to the bloodstream group.

### Differentially expressed genes, up-regulated in bloodstream forms

At the 0.1 FDR, 72 genes have been identified as being up-regulated in bloodstream forms. Thirty two of these were hypothetical genes of unknown function (Additional file 4), four of which (Tb09.211.3955, Tb10.389.0720, Tb11.01.3580, Tb10.07.4080) have been previously identified in *T. b. brucei *as being up-regulated in bloodstream forms in a proteome study[40] and one (Tb927.5.310) by microarray analysis[34]. The identification of these possible coding sequences as being up-regulated in bloodstream forms indicates that they have been correctly classed as genes in the *T. b. brucei *genome confirming the gene model used for their identification.

As expected, a large proportion of genes (22/72) that were differentially regulated at the 0.1 FDR level were associated with antigenic variation; the VSG genes, and related expression site associated genes (ESAGs), both of which were expressed at very high levels in bloodstream forms [41-43]. The VSGs are the major surface coat proteins unique to bloodstream forms, which are encoded by members of a large gene family, although only one VSG gene is expressed at any one time. The VSG gene repertoire is extremely diverse with very few VSG gene sequences being shared between strains[44]. In this analysis, there were three predominant VSG genes that together represent 66% of the VSG tags that uniquely aligned to the reference transcriptome. They aligned to Tb927.8.240, Tb11.21.0001 and Tb09v4.0039 pseudogenes in the TREU 927 reference transcriptome. One hundred and two other VSG genes that uniquely aligned the reference transcriptome were also expressed within this cell population at low levels. It is likely that there were many other VSG gene tags present in the library that did not align to the reference transcriptome, due to strain specific nature of repertoire of VSG genes. According to current dogma, only one expression site, and therefore only one VSG gene, is active in any one trypanosome cell[45], this suggests that there were multiple populations of cells expressing different VSG genes within the bloodstream form cultures analysed. An alternative explanation would be that there were low levels of multiple VSG mRNAs being expressed at the one time in one cell.

The difference in metabolism between the two different life-cycle stages due to the change in the main energy source, from glucose in the bloodstream to proline in procyclic forms[46], was reflected in the up-regulation in bloodstream forms of the THT1- gene encoding a glucose transporter, (Tb10.6k15.2040), a gene encoding a putative glycerol uptake protein (Tb10.61.0380) and the ALD gene encoding fructose-bisphosphate aldolase (Tb10.70.1370), consistent with previous microarray or proteome studies[2,34,40]. Other genes that were up-regulated in bloodstream forms include those involved in intracellular protein transport or modification, i.e. the P67 gene that encodes a lysosomal/endosomal membrane protein (Tb927.5.1810), NsF gene that encodes the vesicular-fusion protein (Tb927.1.1560) and the GPI-PLC gene that encodes glycosylphosphatidylinositol-specific phospholipase C (Tb927.2.6000).

The gene Tb927.2.6180 encoding a putative iron/ascorbate oxidoreductase family protein has not been previously identified as being up-regulated in bloodstream forms using microarray analysis[2,5,34], although it did appear to be up-regulated in bloodstream forms at the protein level[40]. Although this gene was detected only at a low level in bloodstream forms (~10 tags per million) and not detected at all in any of the procyclic form libraries, it was still possible to identify a highly significant difference in transcript abundance, illustrating the sensitivity of digital tag technology to detect differences even for low-abundance transcripts. Similarly, three genes encoding leucine-rich repeat proteins that were up-regulated in bloodstream forms were either not detected at all in the procyclic form libraries (Tb927.8.530 and Tb11.02.1564) or at extremely low levels (Tb927.3.580).

Other genes that were up-regulated in bloodstream forms at the 0.1 FDR include genes that encode dynein heavy chain proteins putatively involved in intraflagellar transport (Tb11.02.0030 and Tb927.4.560), the chaperone protein, DNAJ (Tb927.4.3980), SUMO, involved in post-translational modification (Tb09.160.0070), glutathionylspermidine synthetase (Tb11.12.0016) and the cysteine peptidase, calpain (Tb927.8.8330), none of which had previously been shown to be up-regulated in bloodstream forms in *T. brucei *by microarray analysis[2,5,34].

By setting a stringent FDR, a small number of well-supported genes were identified in this study, however this would undoubtedly exclude a number of genuinely differentially expressed genes, some of which were previously identified in other studies as being differentially expressed. For comparison, we have taken these previously identified differentially expressed genes described in the microarray studies of Koumandou *et al.*[2] and Brems *et al.*[34] and compared them, where possible, with the DGE data in this analysis (Additional file 6). This shows that the vast majority of genes that were previously identified as being up-regulated in bloodstream forms in *T. b. brucei*, were also found to be up-regulated in the related parasite, *T. b. gambiense*, albeit with approximately half of these genes having a *P*-value greater than 0.01.

### A differentially expressed cluster of genes

**Figure 3:**
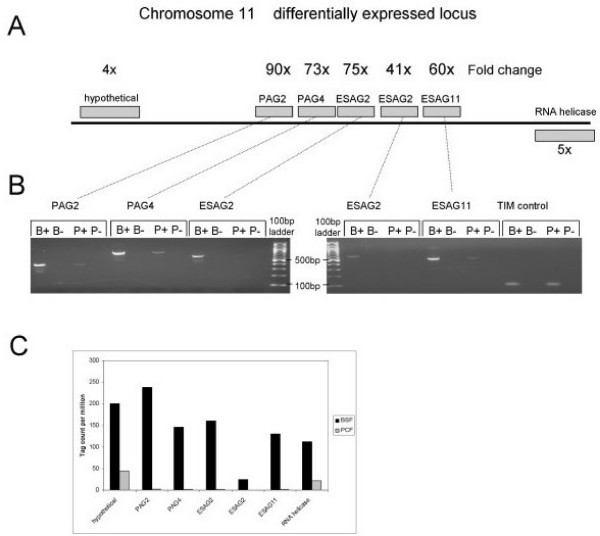
**Genomic context of a differentially expressed gene cluster**. **A **Five contiguous genes are shown with their average fold up-regulation in expression in bloodstream forms. **B **Expression of each gene shown by reverse transcriptase-PCR for bloodstream (B) and procyclic (P) stage parasites, both with (+) and without (-) reverse transcriptase. The triosephosphate isomerase (TIM) gene was used as a constitutively expressed control. **C **Histogram of normalised tag counts (per million tags) showing the abundance of tags from genes in this cluster.

Of the 72 genes that were up-regulated in bloodstream forms above the 0.01 FDR threshold, five contiguous genes (Tb11.01.6210, Tb11.01.6220, Tb11.01.6230, Tb11.01.6240 and Tb11.01.6250) were up-regulated between 41 and 90-fold (Table 2, Figure 3a). These genes, located at a convergent strand switch region at an internal site on chromosome 11, were three ESAGs (two copies of ESAG2 and one ESAG11) and two procyclin associated genes (PAG2 and PAG4). Although most ESAGs are expressed from telomeric expression sites [41-43], there is evidence that some ESAGs can also be expressed from chromosome internal regions of the genome[47]. The identification of two PAG genes that were highly expressed in bloodstream forms compared to procyclic forms was somewhat surprising, as related genes in this multi-gene family were more highly expressed in the procyclic forms in *T. brucei *[48]. Indeed, copies of PAG2 and PAG4 found on chromosome 10 have been shown to be expressed from a polycictronic unit containing the procyclic surface coat genes, EP1 and EP2procyclin[49], which were highly expressed in procyclic forms (up-regulated in procyclics 250-fold, see following section). The functions of the PAG proteins are unknown but there is strong amino acid similarity to the transferrin binding proteins, ESAG6 and ESAG7[50]. In order to verify that these genes were up-regulated in bloodstream forms, RT-PCR was performed for each gene in turn, using primers designed to unique regions of each gene. The RT-PCR clearly confirmed the differential expression of all five genes (Figure 3b). RNAseq data, released prior to publication, for the related sub-species *T. b. brucei *also clearly showed the steady-state RNA levels of these genes was far greater in bloodstream forms than procyclics[35].

**Table 2:** List of putative differentially expressed gene clusters

Up-regulated in bloodstream forms			
**Gene Accession ID**	**Protein ID**	***P*-value**	**average fold change BSF/PCF**

differentially expressed gene cluster 1			
Tb11.01.6210	procyclin-associated gene 2-like protein, putative	0.00000	89.74
Tb11.01.6220	procyclin-associated gene 4 (PAG4) protein, putative	0.00000	73.35
Tb11.01.6230	expression site-associated gene 2 (ESAG2) protein, putative	0.00000	74.77
Tb11.01.6240	expression site-associated gene (ESAG) protein, putative	0.00001	40.70
Tb11.01.6250	expression site-associated gene (ESAG) protein, putative	0.00000	59.76
			
putative differentially expressed gene cluster 2			
Tb10.70.1280	hypothetical protein, conserved	0.00014	17.69
Tb10.70.1290	hypothetical protein, conserved	0.00000	94.86
Tb10.70.1300	procyclin-associated gene 2 (PAG2) protein, putative	0.00185	14.05
Tb10.70.1310	procyclin-associated gene 1 (PAG1) protein, putative	0.00000	122.36
			
putative differentially expressed gene cluster 3			
Tb927.1.4880	hypothetical protein, unlikely	0.00025	22.72
Tb927.1.4890	expression site-associated gene 2 (ESAG2) protein, putative	0.00000	634.48
Tb927.1.4900	expression site-associated gene 11 (ESAG11) protein, putative	0.00017	21.92
			
putative differentially expressed gene cluster 4			
Tb927.1.5100	expression site-associated gene 2 (ESAG2) protein, putative	0.00000	270.71
Tb927.1.5110	expression site-associated gene 11 (ESAG11) protein, putative	0.14722	2.80
Tb927.1.5120	expression site-associated gene (ESAG) protein, putative	0.00005	38.10
Tb927.1.5130	hypothetical protein, unlikely	0.57653	1.17
Tb927.1.5160	hypothetical protein	0.00005	23.47
Tb927.1.5180	hypothetical protein	0.00003	29.84

**Up-regulated in procyclics**			

**Gene Accession ID**	**Protein ID**	***P*-value**	**average fold change PCF/BSF**

putative differentially expressed gene cluster 5			
Tb927.1.2230	calpain-like protein fragment, putative	0.00007	4.13
Tb927.1.2240	hypothetical protein, unlikely	0.00000	7.92
Tb927.1.2250	hypothetical protein, unlikely	0.00010	4.04

Differentially expressed gene clusters were defined as two or more contiguous genes, which were significantly differentially expressed at the 0.2 FDR. The gene accession identification number, protein description and average fold change is given for each gene.

Neighbouring genes within a polycistronic transcription unit are usually regulated independently due to variable mRNA stability, largely regulated by sequences in the 3'UTR of the mRNA. Such post-transcriptional regulation of gene expression has become a distinguishing feature of *T. b. brucei *and the basis of much research[31,51]. Here five consecutive genes were co-regulated and highly expressed in bloodstream form trypanosomes. These genes are situated next to a region where neighbouring polycistronic units meet, termed a strand switch region. Interestingly, divergent and convergent strand switch regions being associated with transcription start and stop sites, respectively[52]. For this gene cluster, the strand switch region is convergent and likely to contain transcription stop sites. There are remarkably short intergenic regions between each of the five ORF in this transcription unit, ranging in size from 12 bp to 203 bp, which could indicate extremely short 3'UTRs of these genes and perhaps the lack of any signals for mRNA degradation. Such highly co-regulated genes are reminiscent of the bloodstream form specific telomeric expression sites, which are unusual in that they are transcribed by RNA polymerase I[32]. Other RNA polymerase I transcription units include the chromosome internal rRNA loci and the EP procyclin and GPEET loci, which are up-regulated in procyclic forms in *T. b. brucei *and which also contain members of the PAG multi-gene family[32]. However, there was no obvious sequences homology between regions upstream of this polycistronic unit and the RNA polymerase I promoters of the procyclin and VSG loci[53].

### Other differentially expressed gene clusters up-regulated in bloodstream forms

Systematic analysis of three or more contiguous differentially expressed genes (with at least two genes being statistically differentially expressed at the 0.2 FDR level of significance) revealed four additional differentially expressed gene clusters (Table 2) that were up-regulated in bloodstream forms and one cluster up-regulated in procyclic forms (Table 2). Gene cluster 2 contains four genes that were up-regulated 14 to 122- fold on chromosome 10. This gene cluster is present in a polycistronic unit that is positioned at a convergent strand switch region and encodes a member of the PAG gene family (PAG1, TB10.70.1310), in a similar manner to the differentially expressed gene cluster on chromosome 11. Two other differentially expressed gene clusters also contain ESAG2 and ESAG11 and, in all cases, the differentially expressed genes appear not to be syntenic with other kinetoplastids and most contain hypothetical genes unique to *T. brucei*. All gene clusters described also show differential mRNA levels in the RNAseq data of *T. b. brucei*[35], except for cluster 5, which was possibly a sub-species differentially expressed gene cluster. What role these genes play in the parasite's life cycle clearly warrants further investigation.

### Differentially expressed genes, up-regulated in procyclics

At the 0.1 FDR level, 25 genes were identified as being up-regulated in procyclic forms (Additional file 4) fewer than were up-regulated in bloodstream forms. The most up-regulated gene identified in procyclic form trypanosomes encoded the procyclic stage specific surface antigen PSSA-2 (Tb10.26.0790) (Additional file 4)[54]. The genes encoding the major procyclic surface coat protein procyclin, EP1 and EP2[48], do not contain *Nla*III sites within the ORF, and so these genes did not appear in the *in silico *derived transcriptome to which the tags were aligned. However a tag matching the 3'UTR of EP2 was identified in the dataset of unaligned tags and was expressed at extremely high level in procyclic forms, up-regulated 250-fold.

A large proportion of the genes up-regulated in procyclic forms were annotated as hypothetical (16/25) providing some insight into their possible role in trypanosome development. Interestingly, several small open reading frames annotated as being unlikely to encode proteins[35] were identified as being differentially expressed and particularly up-regulated in procyclic forms (9 out of 25 at the FDR threshold of 0.1), suggesting the gene annotation is correct.

Known developmentally regulated mRNAs involved in mitochondrial biogenesis and metabolism in procyclic forms that were found to be differentially expressed at the protein level were identified in the present analysis, GMDH (glycosomal malate dehydrogenase, Tb10.61.0980) and PPDK (pyruvate phosphate dikinase, Tb11.02.4150)[34,55]. While many of the cytochrome oxidase subunit genes (Tb11.02.4485, Tb09.160.1820, Tb927.7.2700 and Tb11.01.4707) were identified as being up-regulated in procyclic forms[34] at the 0.2 FDR, they fall just below the stringent 0.1 FDR threshold (Additional file 4). Other genes up-regulated in procyclic forms include TBTS (trans-sialidase, Tb927.7.6850), an amino acid transporter gene (Tb11.02.4520), a gene encoding a calpain-like protein (Tb927.1.2230) and the surface protein gene, CRAM (Tb10.6k15.3510), which have all been previously identified as being up-regulated in procyclic form trypanosomes [2,34,56,57].

Several other genes identified in the present study as being up-regulated in procyclic forms were not identified in previous studies at the mRNA level, including a putative UDP-Gal or UDP-GlcNAc-dependent glycosytransferase gene (Tb927.4.5240), although this was found to be up-regulated in procyclics in a comparative proteome study[40]. Interestingly there are 7 tandemly repeated copies of this gene, but it is only the last gene in the array that was up-regulated in procyclic forms, the rest being up-regulated approximately three-fold in bloodstream forms (although not significant at the 0.1 FDR). Intriguingly, while the other genes in the tandem array share near-identical 3'UTR sequences, Tb927.4.5240 has a very different 3'UTR, consistent with the hypothesis that the 3'UTR contains regulatory elements that determine differential gene expression[29].

Previous analysis of the tandemly repeated gene cluster of phosphoglycerol kinase gene copies (PGKA, PGKB and PGKC), have indicated that each gene is regulated differently in the polycistronic unit, with PGKA being constitutively expressed, PGKB being procyclic form specific and PGKC being bloodstream form specific [58-60]. Analysis of the expression patterns of these genes using DGE indicated that very few unique tags aligned to the ORF of these genes, partly due to the similarity in gene sequence between all three PGK gene copies reducing the number of informative tags. In addition, the majority of tags for PGKC aligned to the 3'UTR, which was not represented in the reference transcriptome. The few unique tags that were generated for these genes did not show any significant difference in tag numbers between life cycle stages. Assuming the expression pattern for these genes is the same in *T.b. gambiense *and *T.b. brucei*, the analysis of this gene family illustrates that this technique only provided relative expression differences for a portion of the differentially expressed transcripts and did not detect all differentially expressed genes.

The most highly expressed gene in procyclic forms was HSP83. Although this gene was also among the top four genes expressed in all bloodstream form libraries, it appeared to be up-regulated in procyclic forms 3.8-fold (although not statistically significant at the 0.2 FDR threshold). Interestingly, HSP83 has been previously identified as being up-regulated in procyclic forms of the pleomorphic strain, *T. b. brucei *927, but not the monomorphic strain *T. b. brucei *Lister 427[34], indicating its differential expression was strain specific. Whole genome sequencing of the STIB 386 strain revealed an estimated 10 copies of this gene in the genome (data not shown), which could explain the high expression levels.

Comparing the previously identified genes that have been described as being up-regulated in procyclic forms in the microarray studies of Koumandou *et al.*[2] and Brems *et al*[34] for *T. b. brucei *with the DGE data in this analysis (Additional file 6), many genes appear to be up-regulated in both types of analysis, confirming previous analysis and suggesting similarities in differentiation pathways between the different sub-species. However there were several genes, which gave contradictory results, i.e. they appeared to be down-regulated (>2 fold) in procyclic forms, albeit not statistically significantly (P > 0.1).

## Conclusions

In this study, the partial transcriptomes of bloodstream and procyclic cells of *T. b. gambiense *were compared using DGE, providing expression data for 7360 genes, constituting 81% of the genome. However this still leaves 19% the genome with no expression information. Some of those genes could be specific to the other life cycle stages not analysed here, such as epimastigotes, while others could be unrepresented due to the limitations of the DGE technique. These limitations include only sampling genes with *Nla*III sites within their open reading frames, an underrepresentation of genes with long 3'UTRs and the inability to align many tags uniquely to the transcriptome. While DGE allows transcription profiling of a large proportion of the genes in the genome, a more comprehensive analysis of the transcriptome could be have been obtained by RNAseq analysis. In addition, allowing for a two base pair mismatch between the tag and the reference transcriptome to take account of sequence differences between the *T. b. brucei *and *T. b. gambiense *genomes could result in misalignment of the tag to the reference transcriptome and could also introduce a bias in some of the analysis. However, this approach is particularly useful when comparing paired samples, in this case different life cycle stages of the same strain and the concordance between the data generated by DGE and that of microarrays, albeit for a different sub-species, indicates that this approach is valid.

DGE has revealed that a larger proportion of genes in *T. b. gambiense *are stage-regulated in contrast to two previous microarray studies on *T. b. brucei*. A recent third microarray study[61] examining gene expression in a time course of parasite differentiation revealed many of the differentially expressed genes identified here were common to all studies. Where this study differs from microarray analysis is in the far greater sensitivity of DGE compared to hybridization-based techniques, which coupled with its high reproducibility means that a larger number of genes that had not previously been identified as being stage-regulated could be identified as being differentially expressed with a high degree of statistical power. The second reason for the difference in gene expression between the DGE analysis and the previous microarray studies is that different strains/sub-species were used. Strains of *T. brucei *can vary naturally in a range of different phenotypes, for example, in terms of virulence, growth, drug resistance and resistance to human serum. Undoubtedly such phenotypic differences will be as a result of different gene expression patterns, although strain-specific differences have been relatively under investigated in this species with the majority of research being focused on a single laboratory-adapted line. The increased power to detect strain-specific differences means that DGE could be a useful tool to examine naturally occurring variable phenotypes, or strains that have been selected for particular phenotypes, and to understand the pathways involved in these processes.

One particular phenomenon revealed by DGE, which had not previously been observed from microarray studies, but which was evident in the pre-publication of RNAseq data of *T. brucei*[35] is the presence of several differentially expressed clusters of genes present on what appears to be the same polycistronic units. These clusters are reminiscent of the RNA polymerase I transcribed polycistronic units involved in the expression of VSG and procyclin genes, which encode the parasite's surface coat. Many of these differentially expressed gene clusters are located in regions of the genome that are unique to *T. brucei*, with no synteny with other kinetoplastids. These regions are also associated with strand switch sites. The strand switch regions of differentially expressed gene clusters identified here are convergent and do not obviously correspond to the possible RNA polymerase II transcription start sites previously identified[52]. It is tempting to speculate that these islands of differentially expressed genes are transcribed by the RNA polymerase that controls transcription of other differentially expressed genes, *i.e*. RNA polymerase I. Indeed four of the gene clusters contain either PAGs or ESAGs, families of genes that are often transcribed by RNA polymerase I in either procyclin or bloodstream expression sites, respectively. However at present it is unclear which RNA polymerase transcribes these life cycle specific gene clusters, although understanding the basis of the control of the differentially expressed gene clusters clearly warrants further investigation to determine if this phenomenon is due to transcript stability of mRNA or transcription rates.

In conclusion we have shown that DGE analysis generates a wealth of data, revealing a large number of hitherto unknown differentially regulated genes and providing new insights into this unusual pathogen.

## Methods

### Sample preparation

Approximately 4.5 × 10^6 ^procyclic cells per library, grown in SDM-79[62] with 10% FBS, were harvested during mid-log phase at 2 × 10^7 ^cells/ml (the saturation density was 5 × 10^7 ^cells/ml). The cells were pelleted at 1100 g for 10 mins at 4°C and immediately re-suspended in RLT buffer (Qiagen RNeasy kit). The cells were disrupted by serial passage through a 26 gauge needle and stored at -80°C before use. Bloodstream form *T. b. gambiense *STIB 386 were grown in HMI9[63] with 20% FBS-plus at 37°C with 5% CO_2 _and harvested at mid-log phase at 4 × 10^5 ^cells/ml (saturation density was 2 × 10^6 ^cells/ml). The cells were pelleted at 900 g for 10 mins at 4°C and immediately re-suspended in RLT buffer. The cells were prepared and stored as above. RNA was isolated from the cells using RNeasy kit (Qiagen) according to manufacturer's instructions, including a DNase treatment step, to ensure that there was no DNA contamination. The RNA was measured for quantity and quality by Nanodrop ND-1000 spectrophotometer and Agilent 2100 Bioanalyzer according to the manufacturer's instructions.

### Digital transcriptomics

Sequence tag preparation was performed with Digital Gene Expression Tag Profile Kit (Illumina), according to the manufacturer's instructions (outlined in Additional file 1). Briefly, 1 μg of total RNA per sample was incubated with oligo-dT beads to capture the polyadenlyated RNA fraction. First-strand and second-strand cDNA was synthesised while the RNA was bound to the beads, and the double stranded product was digested with restriction enzyme *Nla*III, which cuts approximately 250 bp upstream of the messenger RNA polyA tail. The GEX adaptor 1, containing a *Mme*I restriction site, was bound to the free 5' end of the cDNA. The restriction enzyme, *Mme*I, was used to cut 21 bp downstream from the recognition site, thus creating a 21 bp unique tag. After digestion, the 21 bp unique tag and adaptor were purified, dephosphorylated, phenol extracted and ligated to the GEX adaptor 2, complementary to the surface-bound amplification primer on the flow cell. The samples were sequenced according to the manufacturer's instructions at The Gene Pool, Edinburgh. Image analysis and base-calling were performed using the Illumina Pipeline, where sequence tags were obtained after quality filtering. The data has been deposited in NCBI's Gene Expression Omnibus[64] and are accessible through GEO series accession number GSE18065 http://www.ncbi.nlm.nih.gov/geo/query/acc.cgi?acc=GSE18065.

### DGE tag annotation

All tags were mapped to the *in silico *generated transcriptome of *T. b. brucei *TREU 927[35], the most closely related fully annotated genome available to the *T. b. gambiense *strain, using MAQ program maq-0.6.8_x86_64-linux[65], allowing for a 2 bp mismatch between the tag and the reference transcriptome. The *in silico *transcriptome did not contain 5' or 3'UTR sequences as these have not been defined in *T. brucei*. Tags that were generated with a poor quality sequencing score were removed from the analysis. A mapping quality score of 40, incorporating sequence quality and ability of the tag to map to one unique site in the transcriptome, was used to identify tags that align uniquely to the reference sequence. The aligned tags will be available in TritrypDB[35]. This study was limited to tags that map to open reading frames only and does not show tags that map to mRNA with long 3'UTRs.

### Statistical analyses

Genes that were differentially regulated between bloodstream and procyclic form trypanosomes were identified using the Rank Product software[39,66]. This non-parametric method ranks genes by degree of up-regulation, assesses the likelihood that a given gene attained its rank by chance, and estimates a false discovery rate (FDR) for all genes. To avoid confusion all genes with differential gene expression are referred to as being up-regulated either in bloodstream or procyclics, although this does not imply a regulatory mechanism.

### Reverse transcriptase PCR

Reverse-transcriptase PCR (RT-PCR) was carried out on selected genes using the same RNA stock as was utilised in the digital expression analysis. Approximately 1 μg of total RNA was treated using TURBO DNase (Ambion) according to the manufacturer's protocol, to remove any DNA. The reverse transcription was carried out using Omniscript RT kit (Qiagen) with 5 μM oligo dT primers. Identical RT reactions without the reverse transcriptase enzyme were carried out as controls to test for genomic DNA contamination. Each PCR reaction was carried out in 10 μl reaction volumes containing the following: 4.5 mM Tris-HCl (pH8.8), 11 mM (NH4)SO4, 4.5 mM MgCl2, 6.7 mM2-mercaptoethanol, 4.4 uM EDTA, 1 mM each of the four deoxyribonucleotide triphosphates, 10 mM each oligonucleotide primer, 0.5 units *Taq *polymerase (ABgene, UK) and 100 ng cDNA. PCR conditions were as follows: 95°C for 50 s, 58°C for 50 s and 65°C for 50 s for 26 cycles. PCR products were separated by electrophoresis on a 1% Seakem agarose gel and visualised by ethidium bromide staining and UV illumination. Primer sequences for each gene were: Tb11.01.6210-A catgctatggggggatggtat, Tb11.01.6210-B actccttgtccgcttcc, Tb11.01.6220-A gaggttggacgagttggt, Tb11.01.6220-B ccacaataccacagagaatac, Tb11.01.6230-A ggtgaggtataagattacacac, Tb11.01.6230-B cgatatgattcggcttctc, Tb11.01.6240-A gacgagtaccataacgctg, Tb11.01.6240-B tatccattatacacaccgtca, Tb11.01.6250-A atggtaatgatcagaaatac, Tb11.01.6250-B tgttttctcatggaggatcc, tim-E tgccgttgagtgggtgaagatagc, and tim-F ctccctgctacctgtctttacatc.

## Authors' contributions

AM and NV conceived and designed the experiments. NV, ST performed the experiments. PJ, NV, UT, DW, AM performed the analysis. AM, PJ, NV wrote the paper. All authors read and approved the final manuscript.

## Supplementary Material

Additional file 1 Outline of the digital gene expression method. Click here for file

Additional file 2 **Tag abundance**. Normalised tag counts for those tags that aligned to single copy genes. Normalised tag counts were calculated by dividing tag counts for each gene with the total number of tags generated for each library and are presented per 100000 transcripts. Click here for file

Additional file 3 **List of all genes analysed by DGE ranked by average fold change in expression of bloodstream forms over procyclic forms**. The gene accession identification number, protein description and average fold change is given for each gene. Click here for file

Additional file 4 **List of differentially expressed genes, below the FDR of 0.2**. The gene accession identification number, protein description and average fold change is given for each gene. Click here for file

Additional file 5 **QQ plot**. Quantile-quantile plots of the *P*-value distribution for tests of up-regulation in the procyclic (PC) and bloodstream (BS) forms. The y = x line shows the expected quantiles of the ordered *P*-values under the null hypothesis of no up-regulation. The number of times the same *P*-value occurs is indicated by the size of the point area. Click here for file

Additional file 6 **Comparison between microarray data and DGE analysis**. Differentially expressed genes identified in Koumandou *et al*[2]and Brems *et al*[34], were compared, where possible, with data generated by DGE. Shaded pink genes indicate where microarray data from *T. b. brucei *agrees with DGE and blue cells where there is disagreement. The gene accession identification number, protein description and average fold change is given for each gene. Click here for file
