# Supplementary material for: Digital gene expression analysis of two life cycle stages of the human-infective parasite, Trypanosoma brucei gambiense reveals differentially expressed clusters of co-regulated genes
Source: BMC Genomics. 2010 Feb 22;11:124. doi: 10.1186/1471-2164-11-124 (PMC2837033; doi:10.1186/1471-2164-11-124)
Supplement: Additional file 1 — Outline of the digital gene expression method. [file 1471-2164-11-124-S1.PPTX]

## Slide 1
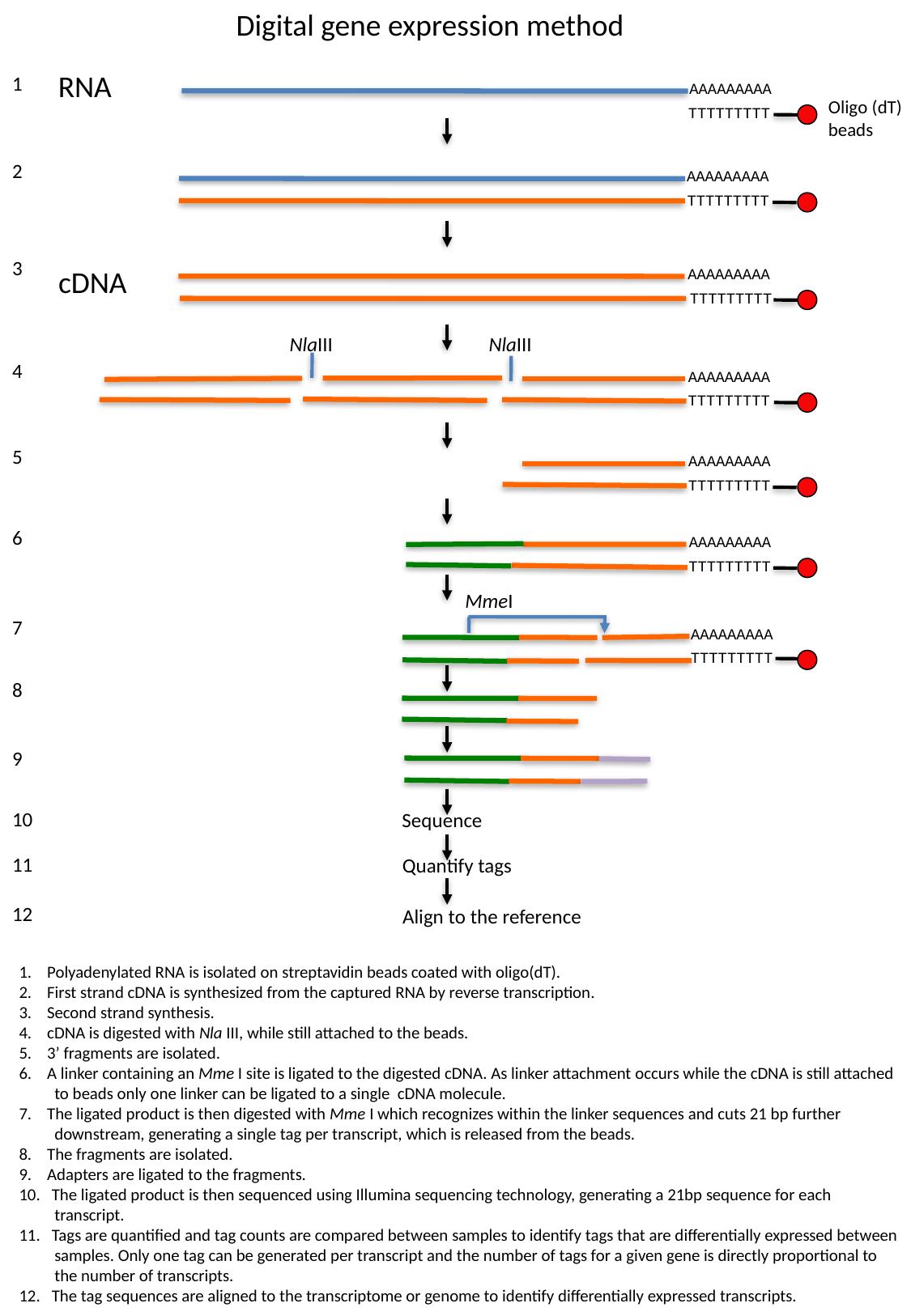

Digital gene expression method
RNA
1
 AAAAAAAAA
Oligo (dT) beads
TTTTTTTTT
2
 AAAAAAAAA
TTTTTTTTT
3
cDNA
 AAAAAAAAA
TTTTTTTTT
NlaIII
NlaIII
4
 AAAAAAAAA
TTTTTTTTT
5
 AAAAAAAAA
TTTTTTTTT
6
 AAAAAAAAA
TTTTTTTTT
MmeI
7
 AAAAAAAAA
TTTTTTTTT
8
9
10
Sequence
11
Quantify tags
12
Align to the reference
1. Polyadenylated RNA is isolated on streptavidin beads coated with oligo(dT).
2. First strand cDNA is synthesized from the captured RNA by reverse transcription.
3. Second strand synthesis.
4. cDNA is digested with Nla III, while still attached to the beads.
5. 3’ fragments are isolated.
6. A linker containing an Mme I site is ligated to the digested cDNA. As linker attachment occurs while the cDNA is still attached to beads only one linker can be ligated to a single cDNA molecule.
7. The ligated product is then digested with Mme I which recognizes within the linker sequences and cuts 21 bp further downstream, generating a single tag per transcript, which is released from the beads.
8. The fragments are isolated.
9. Adapters are ligated to the fragments.
10. The ligated product is then sequenced using Illumina sequencing technology, generating a 21bp sequence for each transcript.
11. Tags are quantified and tag counts are compared between samples to identify tags that are differentially expressed between samples. Only one tag can be generated per transcript and the number of tags for a given gene is directly proportional to the number of transcripts.
12. The tag sequences are aligned to the transcriptome or genome to identify differentially expressed transcripts.
