# Supplementary figures and images for: Digital gene expression analysis of two life cycle stages of the human-infective parasite, Trypanosoma brucei gambiense reveals differentially expressed clusters of co-regulated genes
Source: BMC Genomics. 2010 Feb 22;11:124. doi: 10.1186/1471-2164-11-124 (PMC2837033; doi:10.1186/1471-2164-11-124)

## Slide 1
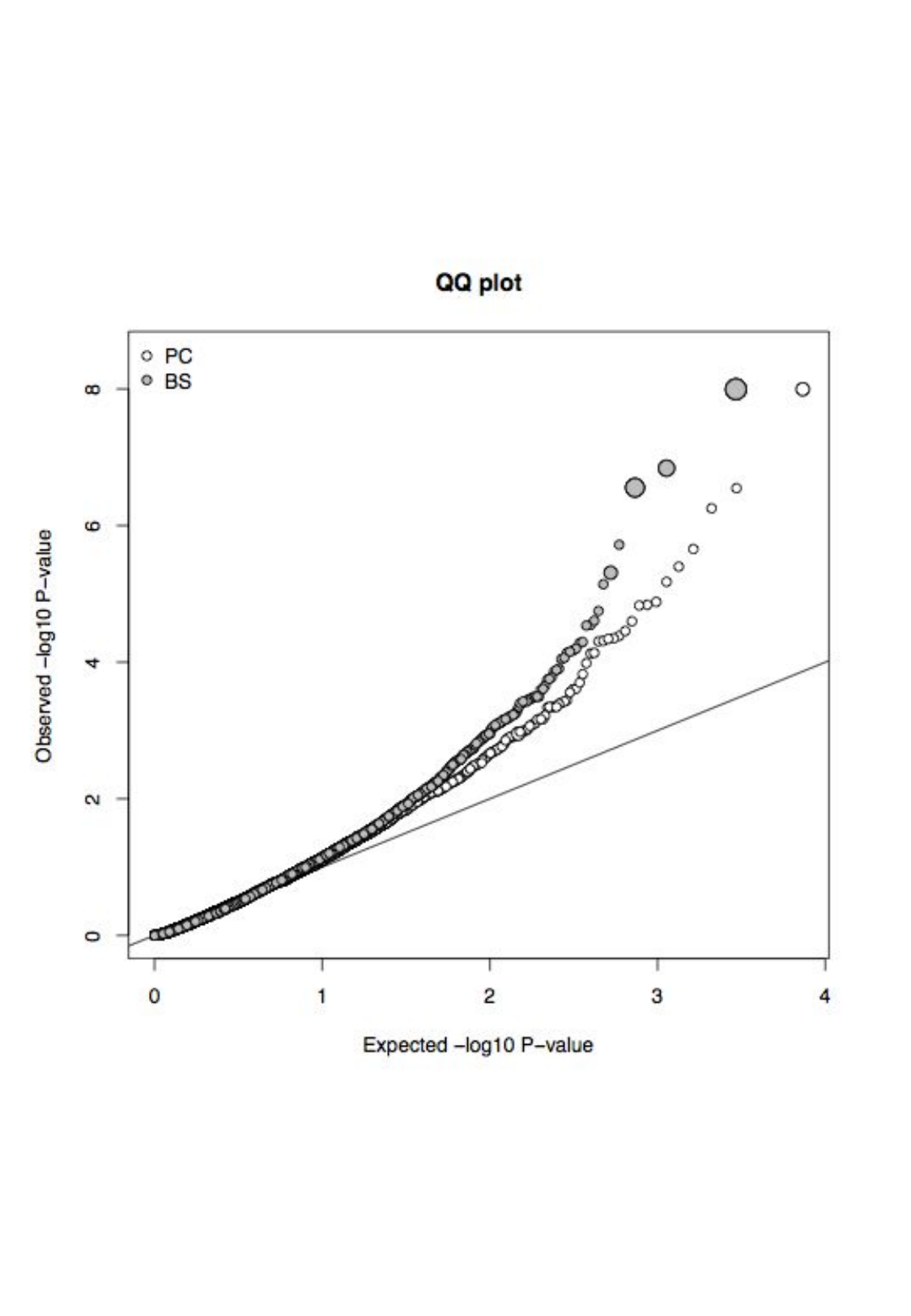

Supplement: Additional file 5 — QQ plot. Quantile-quantile plots of the P-value distribution for tests of up-regulation in the procyclic (PC) and bloodstream (BS) forms. The y = x line shows the expected quantiles of the ordered P-values under the null hypothesis of no up-regulation. The number of times the same P-value occurs is indicated by the size of the point area. [file 1471-2164-11-124-S5.PPT]
