# Supplementary material for: Digital gene expression analysis of two life cycle stages of the human-infective parasite, Trypanosoma brucei gambiense reveals differentially expressed clusters of co-regulated genes
Source: BMC Genomics. 2010 Feb 22;11:124. doi: 10.1186/1471-2164-11-124 (PMC2837033; doi:10.1186/1471-2164-11-124)
Supplement: Additional file 6 — Comparison between microarray data and DGE analysis. Differentially expressed genes identified in Koumandou et al[2]and Brems et al[34], were compared, where possible, with data generated by DGE. Shaded pink genes indicate where microarray data from T. b. brucei agrees with DGE and blue cells where there is disagreement. The gene accession identification number, protein description and average fold change is given for each gene. [file 1471-2164-11-124-S6.DOC]

Additional file 6 Comparison of genes identified as differentially regulated by microarray analysis of *T. b. brucei* with DGE data from *T. b. gambiense.*

| up in procyclics |  |  |  |  | |
| --- | --- | --- | --- | --- | --- |
| Gene Accession ID | Protein ID | *P*-value | average fold change- PCF/BSF | Reference | |
|  |  |  |  |  |  |
| Tb10.6k15.3510 | cysteine-rich, acidic integral membrane protein precursor | 0.0000 | 9.01 | 2 | 34 |
| Tb927.7.6850 | trans-sialidase | 0.0000 | 8.55 | 2 | 34 |
| Tb11.02.4150 | pyruvate phosphate dikinase | 0.0000 | 7.22 |  | 34 |
| Tb10.61.0980 | glycosomal malate dehydrogenase | 0.0000 | 6.90 |  | 34 |
| Tb927.3.4500 | Fumarate hydratese, putative | 0.0004 | 4.98 |  | 34 |
| Tb09.160.1820 | cytochrome oxidase subunit V | 0.0004 | 4.82 |  | 34 |
| Tb10.389.1480 | cytosolic nonspecific dipeptidase, putative | 0.0021 | 4.48 |  | 34 |
| Tb927.7.2700 | NADH-cytochrome b5 reductase, putative | 0.0007 | 4.48 |  | 34 |
| Tb927.1.2230 | calpain-like protein fragment, putative | 0.0001 | 4.13 |  | 34 |
| Tb927.2.4210 | glycosomal phosphoenolpyruvate carboxykinase | 0.0013 | 3.93 | 2 | 34 |
| Tb10.26.1080 | heat shock protein 83 | 0.0030 | 3.77 |  | 34 |
| Tb11.02.1480 | mitochondrial processing peptidase alpha subunit, putative | 0.0061 | 3.19 | 2 |  |
| Tb10.6k15.1350 | pteridine transporter, putative | 0.0064 | 3.06 |  | 34 |
| Tb09.160.5480 | purine nucleoside transporter | 0.0046 | 3.01 |  | 34 |
| Tb09.160.4380 | succinate dehydrogenase, putative | 0.0279 | 2.88 |  | 34 |
| Tb11.02.0580 | vesicular protein trafficking mediator, putative | 0.0019 | 2.79 | 2 |  |
| Tb10.6k15.2020 | glucose transporter | 0.0090 | 2.76 |  | 34 |
| Tb927.4.4620 | cytochrome oxidase subunit VIII | 0.0082 | 2.75 |  | 34 |
| Tb927.3.1380 | ATP synthase beta chain, mitochondrial precursor | 0.0261 | 2.60 |  | 34 |
| Tb927.4.4730 | amino acid transporter, putative | 0.0057 | 2.50 |  | 34 |
| Tb11.02.2310 | prostaglandin f synthase | 0.0326 | 2.36 |  | 34 |
| Tb927.8.7120 | farnesyltransferase, putative | 0.0415 | 2.35 | 2 |  |
| Tb927.7.4390 | threonine synthase, putative | 0.0187 | 2.29 |  | 34 |
| Tb09.211.4700 | reiske iron-sulfur protein , mitochondrial precursor | 0.0142 | 2.17 |  | 34 |
| Tb10.389.0330 | UTP-glucose-1-phosphate uridylyltransferase 2, putative | 0.0163 | 2.02 | 2 |  |
| Tb927.3.1410 | cytochrome oxidase subunit VII | 0.0147 | 1.98 |  | 34 |
| Tb11.01.7880 | microtubule-associated protein | 0.0809 | 1.95 | 2 | 34 |
| Tb11.01.7880 | microtubule-associated protein | 0.0809 | 1.95 |  | 34 |
| Tb927.3.590 | adenosine transporter, putative | 0.0849 | 1.92 |  | 34 |
| Tb927.3.1370 | 40S ribosomal protein S25, putative | 0.0427 | 1.91 | 2 |  |
| Tb927.8.4010 | flagellum-adhesion glycoprotein | 0.0371 | 1.79 | 2 | 34 |
| Tb927.5.1060 | mitochondrial processing peptidase, beta subunit, putative | 0.0499 | 1.67 |  | 34 |
| Tb927.1.2310 | hypothetical protein | 0.0216 | 1.63 |  | 34 |
| Tb927.4.2020 | mu-adaptin 3, putative | 0.1121 | 1.57 | 2 |  |
| Tb09.160.4310 | glutamate dehydrogenase | 0.0595 | 1.55 |  | 34 |
| Tb09.211.4760 | metacaspase 5, putative | 0.1448 | 1.50 | 2 |  |
| Tb11.01.8120 | hypothetical protein, conserved | 0.0943 | 1.48 | 2 |  |
| Tb927.7.2640 | hypothetical protein, conserved | 0.1446 | 1.47 | 2 | 34 |
| Tb10.100.0130 | peroxin 14, putative | 0.1232 | 1.45 | 2 |  |
| Tb11.01.2540 | hypothetical protein, conserved | 0.0433 | 1.39 | 2 |  |
| Tb11.01.4702 | cytochrome oxidase subunit X | 0.0010 | 1.34 |  | 34 |
| Tb11.03.0940 | elongation factor, putative | 0.2705 | 1.28 |  | 34 |
| Tb927.1.580 | phosphate-repressible phosphate permease, putative | 0.1683 | 1.25 |  | 34 |
| Tb927.1.3110 | soluble N-ethylmaleimide sensitive factor (NSF) attachment protein, putative | 0.2954 | 1.16 | 2 |  |
| Tb11.03.0090 | ribokinase, putative | 0.1207 | 1.11 | 2 |  |
| Tb10.6k15.2060 | phosphatidylinositol 3 kinase, putative | 0.3220 | 1.10 | 2 |  |
| Tb10.61.2880 | aconitase | 0.1825 | 1.10 |  | 34 |
| Tb927.5.2570 | translation initiation factor, putative | 0.3715 | 1.09 |  | 34 |
| Tb11.01.3550 | 2-oxoglutarate dehydrogenase E2 component, putative | 0.2852 | 1.05 |  | 34 |
| Tb927.4.3950 | cytoskeleton-associated protein CAP5.5, putative | 0.4066 | 1.05 | 2 | 34 |
| Tb927.8.4950 | kinesin, putative | 0.3448 | 0.96 |  | 34 |
| Tb927.3.3020 | actin-like protein, putative | 0.2245 | 0.91 | 2 |  |
| Tb09.211.2150 | poly(A)-binding protein 1 | 0.4703 | 0.88 | 2 |  |
| Tb11.01.8290 | ATP-dependent DEAD/H RNA helicase, putative | 0.4372 | 0.87 |  | 34 |
| Tb927.7.6000 | hypothetical protein, conserved | 0.4256 | 0.85 |  | 34 |
| Tb09.211.4070 | GPI anchored protein | 0.4465 | 0.85 |  | 34 |
| Tb11.0310 | hypothetical protein | 0.4465 | 0.85 |  | 34 |
| Tb927.7.5950 | hypothetical protein, conserved | 0.2540 | 0.85 |  | 34 |
| Tb927.7.1550 | RNA-editing complex protein | 0.1260 | 0.81 |  | 34 |
| Tb927.8.1790 | hypothetical protein, conserved | 0.0740 | 0.80 |  | 34 |
| Tb09.244.2620 | NADH-ubiquinone oxidoreductase complex I subunit, putative | 0.3804 | 0.79 |  | 34 |
| Tb11.01.1740 | 2-oxoglutarate dehydrogenase E1 component, putative | 0.4521 | 0.78 |  | 34 |
| Tb927.4.1110 | hypothetical protein, conserved | 0.3017 | 0.76 |  | 34 |
| Tb927.1.720 | phosphoglycerate kinase | 0.3842 | 0.74 | 2 |  |
| Tb11.47.0022 | hypothetical protein, conserved | 0.2280 | 0.73 | 2 |  |
| Tb927.3.3190 | serine/threonine-protein kinase, putative | 0.6029 | 0.72 | 2 |  |
| Tb927.4.4350 | hypothetical protein, conserved | 0.6470 | 0.69 | 2 |  |
| Tb11.02.5410 | ubiquitin activating enzyme, putative | 0.1178 | 0.69 |  | 34 |
| Tb11.02.5250 | histone H2B variant, putative | 0.7191 | 0.65 | 2 |  |
| Tb10.6k15.3250 | succinyl-CoA ligase [GDP-forming] beta-chain, putative | 0.7100 | 0.63 |  | 34 |
| Tb927.1.1690 | RNA-editing complex protein | 0.6613 | 0.62 |  | 34 |
| Tb927.7.3550 | hypothetical protein, conserved | 0.5892 | 0.60 | 2 |  |
| Tb927.3.4020 | phosphatidylinositol 4-kinase alpha, putative | 0.7367 | 0.59 | 2 |  |
| Tb11.01.3110 | heat shock protein 70 | 0.2782 | 0.58 | 2 |  |
| Tb10.70.4600 | GTP-binding protein, putative | 0.8161 | 0.53 |  | 34 |
| Tb11.02.0290 | succinyl-coA:3-ketoacid-coenzyme A transferase, mitochondrial precursor, putative | 0.4102 | 0.50 |  | 34 |
| Tb11.01.7800 | nucleoside diphosphate kinase | 0.1627 | 0.40 | 2 |  |
| Tb10.61.0870 | SNARE protein, putative | 0.8293 | 0.36 | 2 |  |
| Tb927.7.2630 | hypothetical protein, conserved | 0.9441 | 0.35 |  | 34 |
| Tb11.01.1650 | signal recognition particle receptor alpha subunit, putative | 0.7361 | 0.35 | 2 |  |
| Tb927.6.710 | dephospho-CoA kinase, putative | 0.6388 | 0.31 | 2 |  |
| Tb927.3.1350 | hypothetical protein, conserved | 0.8607 | 0.30 |  | 34 |
| Tb10.406.0240 | kinetoplastod-specific dual specificity phosphatase, putative | 0.7573 | 0.29 | 2 |  |
| Tb927.7.2970 | ATP-dependent DEAD/H RNA helicase, putative | 0.8851 | 0.28 |  | 34 |
| Tb927.7.7220 | hypothetical protein, conserved | 0.9335 | 0.28 |  | 34 |
| Tb927.7.1460 | hypothetical protein, conserved | 0.9927 | 0.24 |  | 34 |
| Tb11.02.2930 | SNF2 DNA repair protein, putative | 0.9953 | 0.23 |  | 34 |
| Tb927.7.5960 | hypothetical protein, conserved | 0.7611 | 0.22 |  | 34 |
| Tb09.244.2660 | hypothetical protein, conserved | 0.9168 | 0.20 |  | 34 |
| Tb927.3.690 | protein kinase, putative | 0.8203 | 0.19 |  | 34 |
| Tb927.7.5930 | hypothetical protein, conserved | 0.9571 | 0.19 |  | 34 |
| Tb09.160.3240 | hypothetical protein, conserved | 0.9914 | 0.17 | 2 |  |
| Tb11.03.0870 | mitochondrial carrier protein, putative | 0.5161 | 0.17 |  | 34 |

| up in bloodstream forms | |  |  |  |  |
| --- | --- | --- | --- | --- | --- |
| Gene Accession ID | Protein ID | *P*-value | average fold change- BSF/PCF | Reference | |
| Tb927.1.1560 | vesicular-fusion protein NsF, putative | 0.0001 | 20.96 | 2 |  |
| Tb927.5.310 | hypothetical protein | 0.0001 | 19.43 | 2 | 34 |
| Tb10.70.2440 | phosphatidylinositol-4-phosphate 5-kinase, putative | 0.0028 | 16.19 | 2 |  |
| Tb10.70.1370 | fructose-bisphosphate aldolase, glycosomal | 0.0008 | 14.08 |  | 34 |
| Tb927.7.4130 | hypothetical protein, conserved | 0.0011 | 13.73 |  | 34 |
| Tb927.2.6000 | glycosylphosphatidylinositol-specific phospholipase C | 0.0009 | 13.02 | 2 | 34 |
| Tb10.6k15.0940 | hypothetical protein | 0.0014 | 12.98 |  | 34 |
| Tb927.2.3320 | 65 kDa invariant surface glycoprotein | 0.0029 | 12.42 | 2 |  |
| Tb927.3.3720 | Golgi vesicular membrane trafficking protein, putative | 0.0321 | 11.98 | 2 |  |
| Tb927.3.4070 | hypothetical protein, conserved | 0.0013 | 11.55 |  | 34 |
| Tb11.02.0730 | metacaspase | 0.0058 | 10.08 |  | 34 |
| Tb10.61.1750 | C-terminal motor kinesin, putative | 0.0013 | 9.37 |  | 34 |
| Tb927.3.4340 | subtilisin-like serine peptidase | 0.0019 | 9.26 | 2 |  |
| Tb927.3.3270 | ATP-dependent phosphofructokinase | 0.0025 | 8.86 |  | 34 |
| Tb10.70.5290 | major surface protease gp63, putative | 0.0048 | 7.46 | 2 |  |
| Tb927.6.3500 | endosomal trafficking protein RME-8, putative | 0.0162 | 7.09 | 2 |  |
| Tb09.244.2430 | BARP protein | 0.0230 | 6.98 | 2 |  |
| Tb927.7.4920 | hypothetical protein, conserved | 0.0030 | 6.66 |  | 34 |
| Tb11.47.0001 | 65 kDa invariant surface glycoprotein-like protein | 0.0151 | 6.66 | 2 | 34 |
| Tb927.4.5310 | serine/threonine-protein kinase A, putative | 0.0119 | 6.51 | 2 |  |
| Tb10.61.1910 | hypothetical protein, conserved | 0.0081 | 6.38 | 2 |  |
| Tb10.61.2680 | pyruvate kinase 1 | 0.0128 | 6.33 | 2 |  |
| Tb927.8.4330 | small GTP-binding protein Rab11 | 0.0358 | 6.33 | 2 |  |
| Tb927.7.5790 | protein disulfide isomerase, putative | 0.0392 | 6.10 | 2 |  |
| Tb09.211.4240 | phosphoinositide-binding protein, putative | 0.0055 | 6.07 | 2 |  |
| Tb927.2.3880 | heterogeneous nuclear ribonucleoprotein H/F, putative | 0.0183 | 5.99 |  | 34 |
| Tb10.6k15.3640 | alternative oxidase | 0.0176 | 5.93 |  | 34 |
| Tb11.01.3805 | microtubule-associated protein | 0.0350 | 5.29 | 2 | 34 |
| Tb11.01.6260 | RNA helicase, putative | 0.0412 | 5.01 | 2 |  |
| Tb09.160.2420 | syntaxin, putative | 0.0565 | 4.63 | 2 |  |
| Tb927.3.3450 | ADP-ribosylation factor-like protein 3A, putative | 0.0698 | 4.41 | 2 |  |
| Tb09.211.3610 | ubiquitin-activating enzyme E1, putative | 0.0361 | 4.19 | 2 |  |
| Tb11.02.1370 | katanin, putative | 0.1176 | 4.19 | 2 |  |
| Tb11.01.6980 | phosphatidylinositol 3-kinase catalytic subunit, putative | 0.0580 | 4.11 | 2 |  |
| Tb927.5.630 | acidic phosphatase, putative | 0.2191 | 3.87 | 2 |  |
| Tb927.5.3220 | signal peptidase type I, putative | 0.0364 | 3.27 | 2 |  |
| Tb927.6.930 | metacaspase MCA3 | 0.0710 | 3.19 | 2 |  |
| Tb927.7.6440 | hypothetical protein, conserved | 0.1561 | 3.16 | 2 |  |
| Tb09.211.3920 | syntaxin, putative | 0.1581 | 3.08 | 2 |  |
| Tb927.4.2070 | antigenic protein, putative | 0.2027 | 2.92 | 2 |  |
| Tb10.70.1130 | hypothetical protein, conserved | 0.2209 | 2.71 | 2 |  |
| Tb927.4.3770 | protein kinase, putative | 0.2362 | 2.70 | 2 |  |
| Tb927.1.3830 | glucose-6-phosphate isomerase, glycosomal | 0.1930 | 2.68 |  | 34 |
| Tb10.70.3090 | hypothetical protein, conserved | 0.2660 | 2.63 |  | 34 |
| Tb11.46.0014 | ubiquitin carboxyl-terminal hydrolase, putative | 0.2546 | 2.55 | 2 |  |
| Tb927.4.2380 | sarcoplasmic reticulum glycoprotein, putative | 0.2490 | 2.54 | 2 |  |
| Tb10.70.5100 | lysosomal alpha-mannosidase precursor, putative | 0.2655 | 2.33 | 2 |  |
| Tb927.6.1780 | mitogen-activated protein kinase, putative | 0.3632 | 2.27 | 2 |  |
| Tb10.70.0590 | small GTPase, putative | 0.2958 | 2.14 | 2 |  |
| Tb927.4.2080 | hypothetical protein, conserved | 0.3966 | 2.14 | 2 |  |
| Tb927.8.8140 | small GTP-binding rab protein, putative | 0.4573 | 2.02 | 2 |  |
| Tb10.70.7410 | vesicle-associated membrane protein, putative | 0.4985 | 1.82 | 2 |  |
| Tb927.6.2370 | ubiquitin-protein ligase, putative | 0.4646 | 1.73 | 2 |  |
| Tb11.01.2640 | hypothetical protein, conserved | 0.5011 | 1.59 | 2 |  |
| Tb11.02.1280 | subtilisin-like serine peptidase | 0.5908 | 1.58 | 2 |  |
| Tb10.389.0480 | vacuolar protein sorting complex subunit, putative | 0.6725 | 1.46 | 2 |  |
| Tb10.61.1090 | histone H3 variant | 0.5850 | 1.43 | 2 |  |
| Tb11.01.3790 | hypothetical protein, conserved | 0.6839 | 1.39 |  | 34 |
| Tb11.01.4460 | hypothetical protein, conserved | 0.5815 | 1.39 | 2 |  |
| Tb927.7.320 | hypothetical protein, conserved | 0.7656 | 1.33 |  | 34 |
| Tb10.61.2490 | protein kinase, putative | 0.6109 | 1.33 | 2 |  |
| Tb10.70.4620 | hypothetical protein, conserved | 0.7427 | 1.33 | 2 |  |
| Tb11.01.6670 | small GTPase | 0.6708 | 1.20 | 2 |  |
| Tb927.7.3250 | expression site-associated gene (ESAG) protein, putative | 0.7512 | 1.08 | 2 |  |
| Tb11.01.6880 | cytosolic coat protein, putative | 0.8138 | 0.97 | 2 |  |
| Tb11.01.3810 | hypothetical protein | 0.8018 | 0.97 |  | 34 |
| Tb927.8.7110 | serine/threonine-protein kinase A, putative | 0.8018 | 0.97 | 2 |  |
| Tb09.211.0680 | CAAX prenyl protease 1, putative | 0.8505 | 0.97 | 2 |  |
| Tb10.70.5230 | hypothetical protein, conserved | 0.8994 | 0.86 |  | 34 |
| Tb10.406.0320 | ARP2/3 complex 16kDa subunit, putative | 0.9760 | 0.65 | 2 |  |
| Tb927.7.190 | thimet oligopeptidase A, putative | 0.9907 | 0.51 | 2 |  |

The gene accession identification number, protein description and average fold change is given for each gene.
